# Influence of acute kidney injury and its recovery subtypes on patient-centered outcomes after lung transplantation

**DOI:** 10.1038/s41598-024-61352-4

**Published:** 2024-05-07

**Authors:** Jin Ha Park, Jae‑Kwang Shim, Mingee Choi, Hyun-Soo Zhang, Na Hyung Jun, Seokyeong Choi, Young-Lan Kwak

**Affiliations:** 1 Department of Anesthesiology and Pain Medicine, Anesthesia and Pain Research Institute, Yonsei University College of Medicine, 50 Yonsei-ro, Seodaemun-gu, Seoul, 03722 Republic of Korea; 2 Department of Preventive Medicine, Yonsei University College of Medicine, Seoul, Republic of Korea; 3 Biostatistics Collaboration Unit, Department of Biomedical Systems Informatics, Yonsei University College of Medicine, Seoul, Republic of Korea; 4 Departments of Anesthesiology and Pain Medicine, National Health Insurance Service Ilsan Hospital, Goyang, Republic of Korea

**Keywords:** Acute kidney injury, Outcomes research, Risk factors, Quality of life, Respiratory tract diseases

## Abstract

This study aimed to investigate the association between acute kidney injury (AKI) recovery subtypes and days alive out of hospital within the first 3 months (DAOH-90) in patients undergoing lung transplantation. Patients who underwent lung transplantation from January 2012 to December 2021 were retrospectively analyzed and stratified into three groups: no-AKI, early recovery AKI (within 7 days), and non-early recovery AKI group. AKI occurred in 86 (35%) of patients, of which 40 (16%) achieved early recovery, and the remaining 46 (19%) did not. The median DAOH-90 was 21 days shorter in the AKI than in the no-AKI (*P* = 0.002), and 29 days shorter in the non-early recovery AKI group than in the no-AKI group (*P* < 0.001). Non-early recovery AKI and preoperative tracheostomy status were independently associated with shorter DAOH-90. The prevalence of CKD (76%), and 1-year mortality (48%) were highest in the non-early recovery AKI group. Postoperative AKI was associated with an adverse patient-centered quality measure for perioperative care, and shorter DAOH-90. The non-early recovery AKI group exhibited the worst prognosis in terms of DAOH-90, CKD progression, and 1-year mortality, highlighting the important role of AKI and early-recovery AKI on both the quality of life and clinical outcomes after lung transplantation.

## Introduction

Lung transplantation is a life-saving therapy for patients who suffer from end-stage lung disease. While pulmonary function dramatically improves after lung transplantation, the quality of life is often limited by exercise intolerance, sedentary behavior, and skeletal muscle weakness^1–3^. If complications ensue in the early postoperative period, however, it leads to a prolonged use of mechanical ventilation and intensive care unit (ICU) stay, resulting in delayed rehabilitation, reduced daily exercise activity, and prolonged hospital stay or rehospitalization up to 3–6 weeks or longer^1,3^. In this regard, days alive out of hospital (DAOH) after lung transplantation is an appropriate patient-centered quality measure of perioperative outcome since it not only combines overall hospitalization, including death, hospital stay and rehospitalization but also has a marked impact on quality improvement^4^.

Regarding the hindrances to postoperative recovery, acute kidney injury (AKI) frequently occurs after lung transplantation, with incidence rates ranging from 40% to as high as 75%, and is strongly associated with increased mortality^5–8^. Furthermore, the influence of AKI on patient outcomes differs depending on the AKI subtype in terms of its recovery pattern after diagnosis, as well as the severity of AKI in various post-surgical patients including those underwent lung transplantation^6,9–13^. Notably, previous studies have focused on long-term mortality or renal function; however, only a few studies have considered the impact of the various AKI subtypes on the quality of patient recovery, such as DAOH in patients who underwent lung transplantation.

This retrospective study aimed to evaluate the association of AKI and AKI recovery types after diagnosis, with DAOH within the first 3 months (DAOH-90), and 1-year mortality, and thereby, understand the role of renal recovery status on patient-centered as well as clinical outcomes after lung transplantation.

## Materials and methods

**Figure 1:**
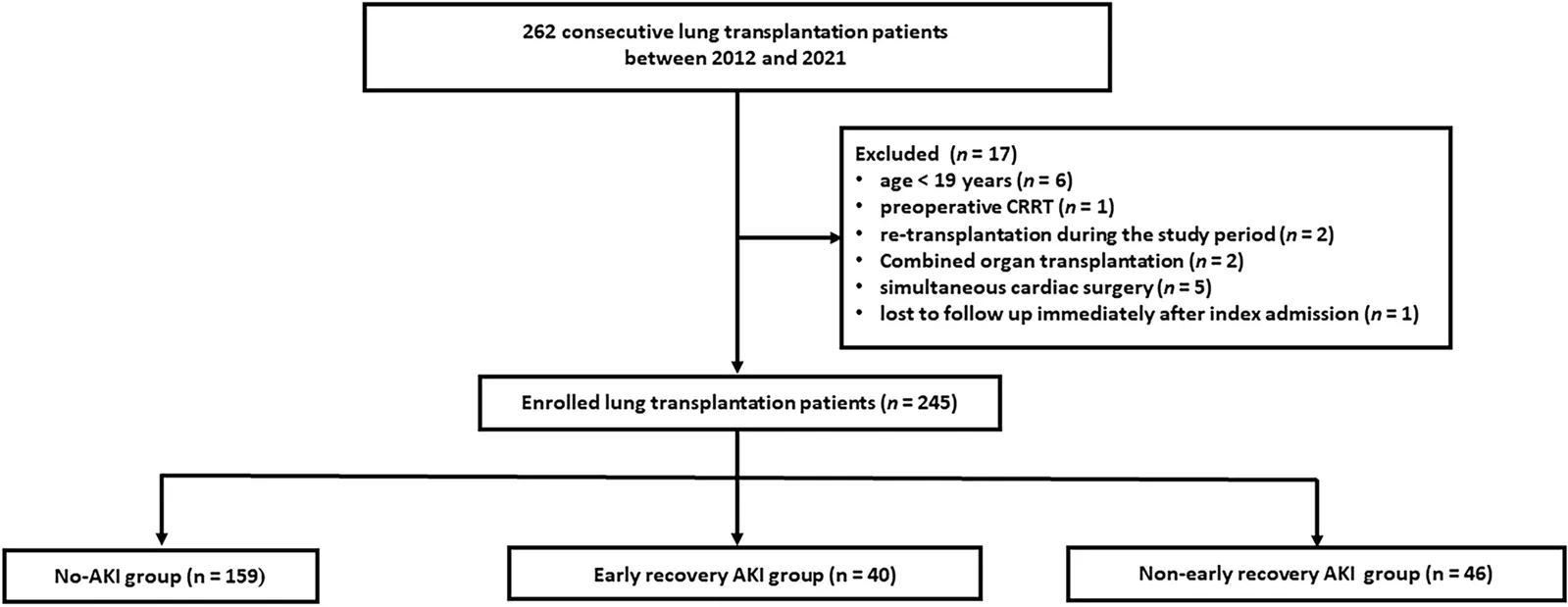
Flowchart of the study population. *AKI* acute kidney injury, *CRRT* continuous renal replacement therapy.

This study was approved by the Institutional Review Board (IRB) of Severance Hospital, Yonsei University College of Medicine (IRB number: 4-2022-1144). The requirement for informed consent was waived owing to the retrospective nature of the study by the Institutional Review Board of Severance Hospital, Yonsei University College of Medicine. This study was conducted in accordance with the principles of the Declaration of Helsinki. We retrospectively reviewed the electronic medical records of 262 patients who underwent lung transplantation between January 2012 and December 2021 at the Severance Hospital, Seoul, Korea. Exclusion criteria were age < 19 years, preoperative renal replacement therapy, re-transplantation during the study period, combined organ transplantation, and simultaneous cardiac surgery. One patient was also excluded because he was lost to follow up. A total of 245 patients were included in this study (Fig. 1).

Patient data were retrospectively retrieved up to February 28, 2023. Preoperative data included patients’ demographics, including age, sex, body mass index, medical history, smoking status, preoperative functional status and laboratory data. Intraoperative data included operative and anesthesia time, fluid balance, transfusion, single or double lung transplantation, and intraoperative extracorporeal membrane oxygenation (ECMO) weaning. Immediate postoperative data included the presence of grade 3 primary graft dysfunction (PGD) within 72 h of lung transplantation, mechanical ventilation day, duration of ECMO support after lung transplantation, and length of hospital and ICU stay. Postoperative day (POD) 7 data included failure to achieve mechanical ventilation weaning, failure to achieve ECMO weaning, tracheostomy rate among patients who did not undergo tracheostomy before lung transplantation, reoperation, and incidence of postoperative complications, such as bronchopleural fistula, pneumonia, and atrial fibrillation, 7 days after surgery. The incidence of AKI and AKI recovery type were assessed. Mid and long-term postoperative data included the incidence of chronic kidney disease (CKD), DAOH-90, and 1-year mortality.

AKI and CKD were determined by changes in serum creatinine levels according to the Kidney Disease Improving Global Outcomes (KDIGO) criteria^14^, and AKI was defined as an increase in serum creatinine concentration to 0.3 mg/dL within 2 days after lung transplantation or a 50% increase within the first 7 postoperative days. Baseline creatinine value was defined as the most recently documented serum creatinine level before lung transplantation. Because serum creatinine level was measured daily during the hospitalization, as well as on every outpatient follow-up visit, serum creatinine levels were retrieved from the electronic medical records up to 1 year after lung transplantation. Urine output criteria were not used because the data were not available. CKD was defined as abnormalities in kidney structure and function of > 3 months. Functional criteria for CKD are glomerular filtrate rate < 60 mL/min/1.73 m^2^.

Early recovery AKI was defined as an absence of AKI within 7 days of the onset of AKI^11,15^. Non-early recovery AKI was defined as all AKI cases not meeting the definition of early recovery AKI^16,17^. Patients with relapsing AKI occurring more than 7 days after early recovery were classified as the early-recovery AKI group. Among patients receiving renal replacement therapy, those who failed to discontinue renal replacement therapy within 7 days after onset of AKI were classified into non-early recovery AKI group, while those who discontinued were classified into the early recovery AKI group.

PGD was graded based on diffuse pulmonary oedema on a chest radiograph and a PaO_2_/FiO_2_ ratio, according to the criteria of the International Society of Heart and Lung Transplant (ISHLT) Working Group^18^.

DAOH was defined as previously described by Myles et al.^19,20^ DAOH-90 was calculated from the total postoperative duration of the index and subsequent hospital stays during POD 90 after surgery. For instance, DAOH-90 was calculated as 90 − (index length of stay [LOS] + subsequent LOS within postoperative 90 days + the length until the day of death before POD 90). If a patient died during the index hospitalization, the DAOH was 0 (zero).

The primary outcome of the study was DAOH-90 after lung transplantation. Specifically, the relationship between AKI recovery types and DAOH-90 was the research question of interest. Therefore, patients were stratified into three groups: patients without AKI (no-AKI), patients with early recovery AKI, and patients with non-early recovery AKI groups. The secondary outcomes were the incidence of CKD and 1-year mortality rate. In addition, patients’ characteristics associated with DAOH-90 were investigated.

### Statistical analysis

Sample sizes of each AKI recovery group, Q-Q plots of normality, and normality tests such as the Kolmogorov–Smirnov test were used to for a nonparametric presentation of the descriptive statistics. Hence, continuous variables were presented as median (interquartile range) or mean (± standard deviation), and categorical variables as N (%). The Kruskal–Wallis test (one-way ANOVA on ranks) was used to compare the group ranks of continuous variables, while the chi-square test, or Fisher’s exact test when needed, was used to compare the group proportions of categorical variables in the three AKI recovery groups. Kaplan–Meier survival curves were constructed to compare 1-year mortality between the AKI recovery groups.

For variables that showed statistically significantly different mean values or proportions between the AKI recovery groups, post-hoc pairwise comparisons were conducted. Specifically, Tukey’s adjustment for continuous variables and Fisher’s exact test with false discovery rate adjustment for categorical variables were performed to verify which pairs among the three AKI groups showed such a difference. For a similar purpose, a random intercept linear mixed model was utilized to determine the pairs of AKI recovery groups with different mean serum creatinine levels over time during the 1-year follow-up period.

Among the independent variables considered for potential association with DAOH-90 in lung transplantation patients, an exposure variable of interest was postoperative AKI. Other independent variables initially considered were patient age, sex, smoking, preoperative tracheostomy, intraoperative ECMO weaning, grade 3 PGD within postoperative 72 h, and postoperative atrial fibrillation within 7 days, based on statistical tests of association with DAOH-90, a priori clinical knowledge, and evidence from previous studies on the prognosis of lung transplantation patients^5,6,9,21^. A correlation analysis among these independent variables revealed a high correlation between sex and smoking and between intraoperative ECMO weaning and grade 3 PGD within postoperative 72 h, which were 0.72 and 0.69, respectively. To prevent multi-collinearity and based on clinical grounds, smoking status and grade 3 PGD within postoperative 72 h were removed from further multivariable analyses.

Quantile regression analysis^22^ was used to investigate the potential risk factors of shorter DAOH-90 among lung transplantation patients, as the bimodal shape of DAOH-90 posed challenges for assuming a parametric distribution. Thus, the τ-th conditional quantile of DAOH-90, given a matrix of independent variables, was modelled as a linear function of the independent variables, for which the regression coefficients, standard errors, and *P*-values were estimated using the *quantreg* package in R^23^. Since the quantile τ ranges from 0 to 1, tests of difference in the estimated regression coefficients by different values of τ were conducted for τ values of 0.25, 0.5, and 0.75, from which non-significant differences were confirmed. Thus, τ = 0.5 was chosen, and the median of DAOH-90 was regressed upon the independent variables considered.

A Sankey flow diagram was also drawn using the *networkD3* package in R^24^. The diagram visualizes patient prognosis trajectories based on AKI status and AKI recovery groups, resulting in different numbers or proportions of CKD and mortality in the study population throughout the 1-year follow-up^25^.

The patient characteristics associated with AKI status and AKI recovery groups (the main exposures of interest) were examined in an exploratory manner. The statistical methods are described in detail in the Supplementary Methods.

All tests were two-sided, and a *P* < 0.05 was considered statistically significant. R 4.1.2 (The R Foundation for Statistical Computing, Vienna, Austria) and SAS 9.4 (Cary, NC, USA) were used for the analyses.

## Results

Of the 245 patients, AKI occurred in 86 patients (35%). Among them, 40 patients recovered early after AKI (early recovery AKI group), and the remaining 46 patients were classified as the non-early recovery AKI group. Patients in the non-early recovery AKI group were more frequently female, more often received tracheostomy before lung transplantation, and were less likely to be smokers than patients in the no-AKI group (Table 1).

**Table 1:** Demographic data.

variables	No-AKI (n = 159)	AKI		
		Early recovery (n = 40)	Non-early recovery (n = 46)	*P* value
Age (years)	58 (50–63)	57 (45–63)	57 (49–64)	0.716
Female	50 (31%)	17 (43%)	24 (52%)^†^	0.028
Height (cm)	165 ± 8	165 ± 9	164 ± 9	0.557
Body mass index (kg/m^2^)	21 (19–24)	21 (18–25)	22 (19–25)	0.878
Smoking history	93 (59%)	20 (50%)	17 (37%)^†^	0.033
Smoking pack year	25 (15–33)	25 (11–40)	30 (20–50)	0.399
Primary diagnosis				
Idiopathic pulmonary fibrosis	99 (62%)	25 (63%)	25 (54%)	0.608
Interstitial lung disease	53 (33%)	16 (40%)	10 (22%)	0.173
Chronic obstructive lung disease	27 (17%)	6 (15%)	3 (7%)	0.210
Graft-versus-host disease	12 (8%)	5 (13%)	5 (11%)	0.547
Connective tissue disease	33 (21%)	9 (23%)	8 (17%)	0.828
Co-morbidities				
Hypertension	30 (19%)	9 (23%)	6 (13%)	0.509
diabetes	34 (21%)	12 (30%)	8 (17%)	0.351
Tuberculosis	56 (35%)	12 (30%)	10 (22%)	0.216
Pulmonary artery hypertension	32 (20%)	7 (18%)	9 (20%)	0.932
Preoperative status				
Hospital admission	92 (58%)	20 (50%)	28 (61%)	0.569
ICU admission	64 (40%)	17 (43%)	23 (50%)	0.500
Tracheostomy	39 (25%)	10 (25%)	21 (46%)^†^	0.017
Home O_2_	148 (93%)	36 (90%)	37 (80%)^†^	0.040
Mechanical ventilation	49 (31%)	13 (33%)	23 (50%)	0.052
ECMO	48 (30%)	11 (28%)	18 (39%)	0.435
EF (%)	65 (61–70)	63 (59–69)	64 (60–74)	0.259
RVSP (mmHg)	47 (38–60)	51 (35–60)	51 (39–71)	0.383
Measured PASP (mmHg)	36 (30–49)	40 (31–46)	34 (30–49)	0.888
6MWT (m)	228 (130–320)	240 (160–315)	230 (125–325)	0.892
FEV1 (%)	41 (30–56)	42 (30–56)	44 (35–57)	0.837
FVC (%)	41 (33–50)	40 (30–54)	39 (33–50)	0.720
Preoperative laboratory values				
Cr (mg/dL)	0.56 (0.48–0.72)	0.52 (0.37–0.64)	0.64 (0.34–0.85)	0.204
eGFR (mL/min/1.73 m^2^)	111 (101–124)	115 (105–131)	104 (92–130)	0.144
Albumin (g/dL)	3.5(3.0–4.0)	3.5 (2.8–4.0)	3.3 (2.8–3.8)	0.152
Platelet (10^3^/μL)	211 (149–276)	229 (164–275)	190 (124–247)	0.161
Lymphocytes (10^3^/μL)	1.4 (0.9–2.0)	1.2 (0.7–2.0)	1.1 (0.7–1.5)	0.199
Neutrophils (10^3^/μL)	7.6 (5.8–10.5)	6.2 (4.9–8.9)	7.9 (4.4–12.2)	0.093
PNI	42 (35–51)	44 (33–50)	38 (31–46)	0.089
NLR	5.4 (3.6–11.1)	5.2 (2.9–9.7)	5.8 (3.4–14.9)	0.704
PLR	157 (100–248)	157 (117–258)	178 (120–233)	0.572
CRP (mg/L)	13 (4–42)	12 (3–32)	16 (3–83)	0.502

Values are median (interquartile range), mean (± standard deviation) or the number of patients.

*AKI* acute kidney injury, *CRP* c-reactive protein, *ECMO* extracorporeal membrane oxygenation, *EF* ejection fraction, *eGFR* estimated glomerular filtration rate, *FEV1* forced expiratory volume in first second, *FVC* forced vital capacity, *IQR* interquartile range, *NLR* neutrophil-to-lymphocyte ratio, *PASP* pulmonary artery systolic pressure, *PLR* platelet-lymphocyte ratio, *PNI* prognostic nutritional index, *RVSP* right ventricular systolic pressure, *6MWT* 6-min walk test.

^†^*P* < 0.05 compared with the no-AKI group.

All patients received cardiopulmonary support during surgery, with the majority undergoing ECMO.The success rate of intraoperative ECMO weaning was significantly lower in the non-early AKI group than in the no-AKI group. The number of patients who received colloid (all patients received 6% hydroxyethylstarch 130/0.4 in a balanced solution, Volulyte^®^; Fresenius Kabi, Bad Homburg, Germany) intraoperatively was higher in the non-early recovery AKI group than in the no-AKI group. Grade 3 PGD within 72 h occurred more frequently in the non-early recovery AKI group than in the no-AKI and early recovery AKI groups. The incidence of failure to achieve weaning from mechanical ventilation and reoperation within POD 7 was higher in the non-early recovery AKI group than in the no-AKI and early recovery AKI groups. The incidence of atrial fibrillation was significantly lower in the no-AKI group than in the early and non-early recovery AKI groups. The length of ICU stay was longer in the non-early recovery AKI group compared to the no-AKI group. The length of hospital stay was longer in the non-early recovery AKI group than in the no-AKI and early recovery AKI groups (Table 2).

**Table 2:** Intraoperative and immediate postoperative data.

Variables	No-AKI (n = 159)	AKI		
		Early recovery (n = 40)	Non-early recovery (n = 46)	*P* value
Intraoperative				
Operative time (min)	374 (338–423)	386 (365–441)	391 (350–448)	0.096
Anesthesia time (min)	470 (430–520)	480 (435–519)	488 (449–529)	0.165
Double LTx	152 (96%)	38 (95%)	45 (98%)	0.757
Support category				0.073
ECMO	157 (99%)	37 (92.5%)	45 (98%)	
CPB	2 (1%)	3 (7.5%)	1 (2%)	
Crystalloid (mL/kg)	110 (87–153)	103 (88–149)	126 (90–179)	0.647
Colloid (mL/kg)	0 (0–10)	0 (0–14)	6 (0–12)	0.108
Use of colloid	55 (35%)	18 (45%)	25 (54%)^†^	0.043
Bleeding (mL)	2000 (1475–3500)	2200 (1400–3500)	2500 (1450–6000)	0.311
Urine output (mL)	1350 (845–2030)	1075 (676–2503)	1168 (604–2543)	0.734
Transfusion				
pRBC	6 (4–8)	6 (4–9)	7 (4–11)	0.135
FFP	3 (0–5)	3 (0–5)	3 (0–6)	0.616
Platelets	5 (0–6)	6 (0–6)	6 (0–12)	0.211
ECMO weaning	99 (62%)	22 (55%)	19 (41%)^†^	0.039
Immediate postoperative data				
Grade 3 PGD_72 h	46 (29%)	11 (28%)	24 (52%)^†‡^	0.009
MV day (d)	4 (2–7)	4 (2–7)	6 (3–11)	0.191
ECMO time (h)	38 (5–145)	40 (5–214)	112 (5–294)	0.207
Postoperative day 7 data				
MV weaning failure	63 (40%)	21 (53%)	34 (74%)^†‡^	< 0.001
Tracheostomy*	16 (13%)	2 (7%)	5 (20%)	0.344
ECMO weaning failure	5 (3%)	1 (3%)	3 (7%)	0.513
Reoperation	18 (11%)	5 (13%)	15 (33%)^†‡^	0.002
BPF	1 (1%)	0 (0%)	1 (2%)	0.485
Pneumonia	120 (76%)	35 (88%)	39 (85%)	0.144
a.fib	11 (7%)	9 (23%)^†^	12 (26%)^†^	< 0.001
Length of stay				
ICU day (d)	7 (5–11)	9 (5–17)	14 (6–24)^†^	< 0.001
Admission day (d)	37 (25–60)	50 (27–92)	69 (33–144)^†‡^	< 0.001

Values are median (interquartile range) or the number of patients.

*a.fib* atrial fibrillation, *AKI* acute kidney injury, *BPF* bronchopleural fistula, *CPB* cardiopulmonary bypass, *ECMO* extracorporeal membrane oxygenation, *FFP* frozen fresh plasma, *ICU* intensive care unit, *IQR* interquartile range, *LTx* lung transplantation, *MV* mechanical ventilation, *PGD* primary graft dysfunction, *PGD_72 h* PGD within 72 h, *pRBC* packed red blood cells.

*The number and percentage of patients who underwent tracheostomy within 7 days after lung transplantation among those who did not undergo tracheostomy before lung transplantation.

^†^*P* < 0.05 compared with the no-AKI group.

^‡^*P* < 0.05 compared with the early recovery AKI group.

**Figure 2:**
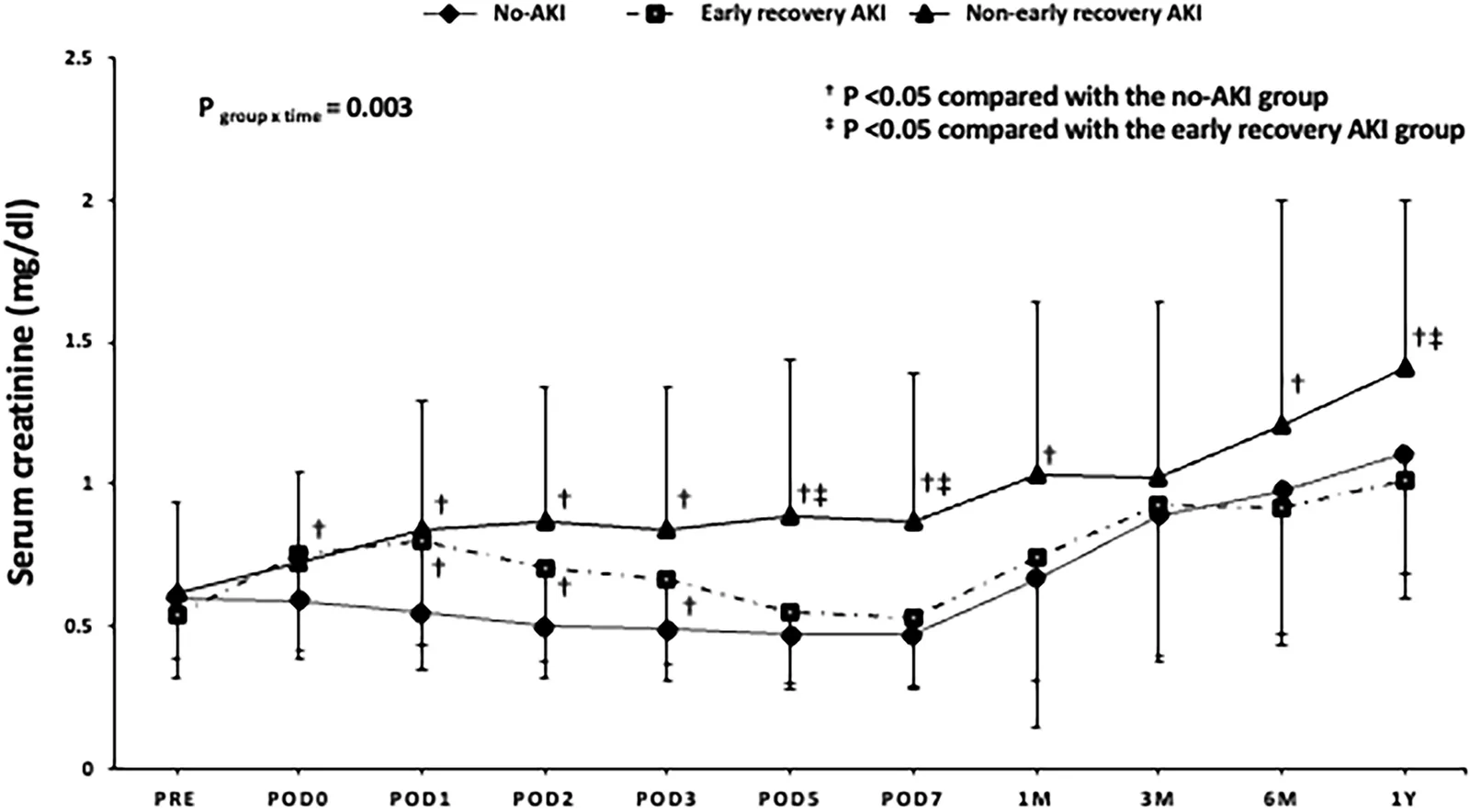
Changes in serum creatinine levels in patients who underwent lung transplantation by AKI recovery group. *AKI* acute kidney injury, *POD* postoperative day.

Serum creatinine levels were higher in the non-early recovery AKI group than in the no-AKI group throughout the 1-year follow-up period (except at POD 0 and 3 months after surgery) and in the early recovery AKI group at POD 5 and 7, and 1 year after surgery. Serum creatinine levels were higher in the early recovery AKI group than in the no-AKI group during POD 0–3, whereas there were no intergroup differences thereafter (Fig. 2).

**Figure 3:**
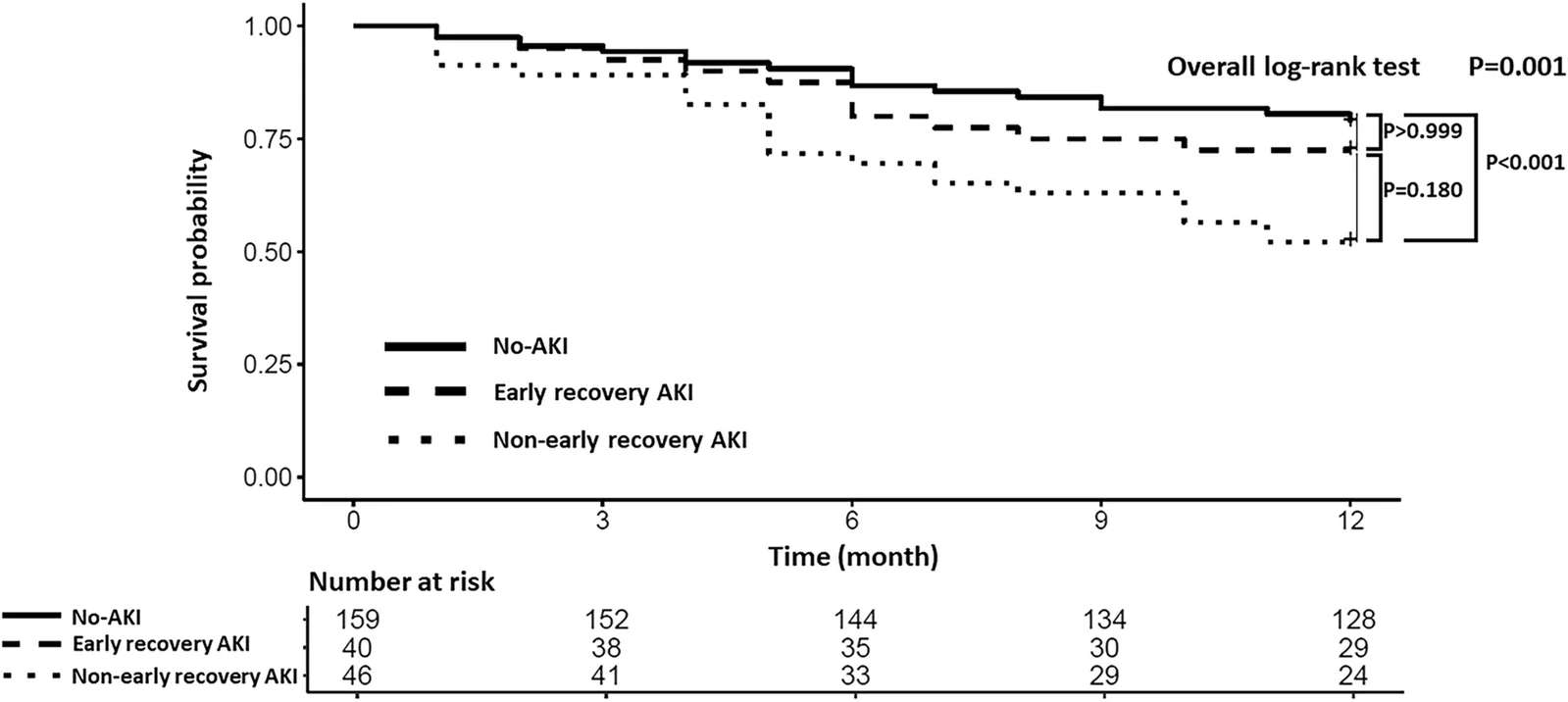
Kaplan–Meier survival curves for 1-year overall survival after lung transplantation. *AKI* acute kidney injury.

Thirty (75%), eight (20%) and two (5%) patients in the early recovery AKI group and 16 (35%), 12 (26%) and 18 (39%) patients in the non-early recovery AKI group developed stage 1, stage 2, and stage 3 AKI, respectively (*P* < 0.001). Among the 20 patients with AKI stage 3, 14 (70%) required renal replacement therapy within 7 days after the onset of AKI. A post-hoc analysis of the difference in the incidence of early recovery according to the AKI stage revealed a difference only between patients with stage 1 and 3 AKI (*P* < 0.001). Differences in the DAOH-90 were found only between patients with stage 1 and 3 AKI (*P* = 0.01) with DAOH-90 of 27, 26, and 0 days in stage 1, stage 2 and stage 3 AKI, respectively. The prevalence of CKD was higher in the non-early recovery AKI group (76%) than in the early recovery AKI group (49%) or the no-AKI group (39%) (both *P* < 0.001). The median DAOH-90 was shorter in the non-early recovery AKI group than in the no-AKI group (*P* < 0.001). The number of patients with DAOH-90 value of 0 was significantly higher in the early and non-early recovery AKI groups (35% and 52%, respectively) than in the no-AKI group (19%, *P* < 0.001). The 1-year mortality rate was significantly higher in the non-early recovery AKI group than in the no-AKI group (Table 3). Kaplan–Meier survival curves confirmed a significantly worse survival at 1 year after lung transplantation in the non-early recovery AKI group than in the no-AKI group (*P* < 0.001, Fig. 3).

**Table 3:** Renal data and mid and long-term outcome.

Variables	No-AKI (n = 159)	AKI		
		Early recovery (n = 40)	Non-early recovery (n = 46)	*P* value
AKI				< 0.001
Stage 1		30 (75%)	16 (35%)	
Stage 2		8 (20%)	12 (26%)	
Stage 3		2 (5%)	18 (39%)	
CKD*	58 (39%)	18 (49%)	31 (76%)^†‡^	< 0.001
DAOH				
DAOH-90 (d)	47 (11–58)	31 (0–55)	0 (0–32)^†^	< 0.001
DAOH-90 of 0	30 (19%)	14 (35%)^†^	24 (52%)^†^	< 0.001
Death				
Death_3m	9 (6%)	3 (8%)	5 (11%)	0.467
Death_1year	34 (21%)	11 (28%)	22 (48%)^†^	0.002

Values are median (interquartile range) or the number of patients.

*AKI* acute kidney injury, *CKD* chronic kidney disease, *DAOH* days alive out of hospital, *DAOH-90* DAOH within 90 days, *DAOH-1y* DAOH within 1 year, *IQR* interquartile range.

*Among survivors until 3 months after lung transplantation.

^†^*P* < 0.05 compared with the no-AKI group.

^‡^*P* < 0.05 compared with the early recovery AKI group.

**Figure 4:**
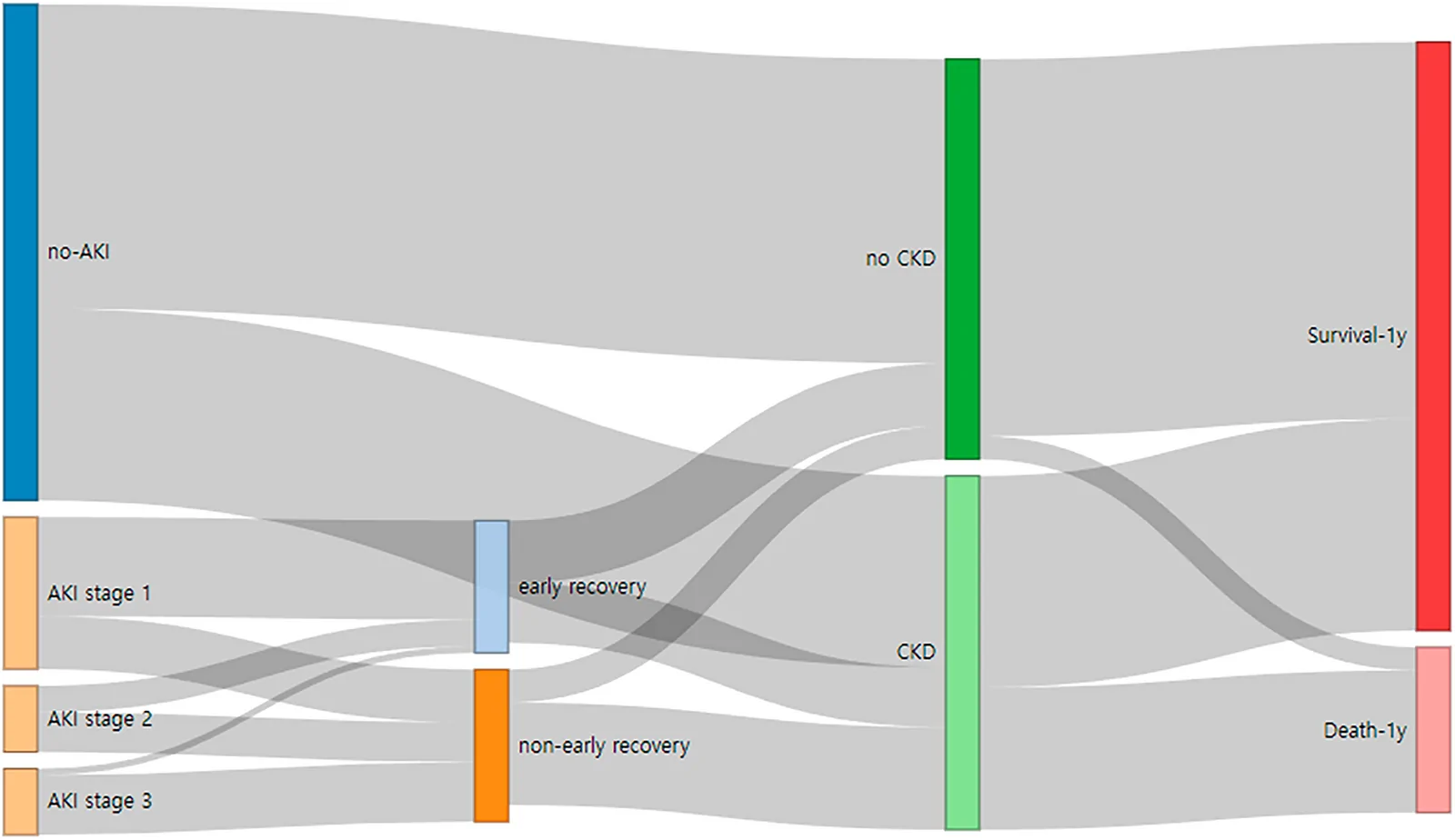
Sankey flow diagram showing prognosis trajectories by AKI status and AKI recovery groups throughout the 1-year of follow-up after lung transplantation. *AKI* acute kidney injury, *CKD* chronic kidney disease.

Unlike its recovery status, AKI stages were not associated with CKD progression (*P* = 0.417) or 1-year mortality rate (*P* = 0.214) (Fig. 4).

Table 4 shows the results of multivariable quantile regression of DAOH-90, by either AKI occurrence or AKI recovery groups. In each model, the median DAOH-90 calculated using regression coefficient was 21 days shorter in patients with AKI than in patients with no-AKI (*P* = 0.002), and 29 days shorter in the non-early recovery AKI group than in the no-AKI group (*P* < 0.001). Patients who underwent tracheostomy before lung transplantation also showed a shorter median DAOH-90.

**Table 4:** Multivariable quantile regression results of DAOH-90 among lung transplantation patients where either AKI occurrence or AKI recovery groups is the main exposure of the interest.

Variables	AKI (Yes/No)			AKI recovery group (0/1/2)*		
	Reg. coeff	SE	*P*-value	Reg. coeff	SE	*P* value
AKI (Yes/No)	− 21.33	6.77	0.002	–	–	–
recovery group: 1 (vs. 0)	–	–	–	− 10.50	9.61	0.276
recovery group: 2 (vs. 0)	–	–	–	− 28.50	6.52	< 0.001
age (in years)	− 0.39	0.21	0.061	− 0.50	0.21	0.018
female	− 2.67	4.87	0.584	− 1.50	4.77	0.754
preoperative tracheostomy status	− 19.91	6.73	0.003	− 15.50	5.94	0.010
intraoperative ECMO weaning	11.15	5.66	0.050	8.50	5.58	0.129
a.fib_7d	− 1.55	9.28	0.868	− 2.50	7.10	0.725

*a.fib* atrial fibrillation, *a.fib_7d* new-onset a.fib within 7d, *AKI* acute kidney injury, *DAOH-90* days alive out of hospital within 90 days, *ECMO* extracorporeal membrane oxygenation, *Reg. coeff.* regression coefficient, *SE* standard error.

*0, no-AKI group; 1, early recovery AKI group; 2, non-early recovery AKI group.

Patient characteristics associated with AKI occurrence were the female sex, preoperative lower neutrophil counts, preoperative mechanical ventilation status, failure to achieve intraoperative ECMO weaning and intraoperative use of colloid (Table S1). Patient characteristics associated with non-early recovery AKI were preoperative tracheostomy status and intraoperative use of colloid (Table S2).

## Discussion

In this single-center retrospective study, the occurrence of AKI was associated with adverse patient-centered mid-term outcome in the AKI group (shorter DAOH-90 by 21 days than in the no-AKI group), which was even worse in the non-early recovery AKI group (shorter DAOH-90 by 29 days than in the no-AKI group). Shorter DAOH-90 was independently associated with non-early recovery AKI and preoperative tracheostomy status. Moreover, the non-early recovery AKI group exhibited the worst prognosis in terms of CKD progression and 1-year mortality rate. In contrast, there was no significant difference in DAOH-90 between the no-AKI and early recovery AKI groups.

Lung transplantation is often associated with poorer health outcomes than other solid organ transplantation. Among various perioperative complications, AKI is one of the most common postoperative complications^5,8^ closely related to short-and long-term prognosis^26^. Prolonged hypoxemia, hemodynamic instability, blood transfusion, inflammatory response, and ischemia–reperfusion injury induced by ECMO or cardiopulmonary bypass have been reported to contribute to AKI development after lung transplantation^5,6^. Consistent with previous reports, in this study, AKI occurred in 35% of the patients, which was significantly associated with the female sex, preoperative mechanical ventilation, and failure to achieve weaning from ECMO intraoperatively. Additionally, colloid use increased the risk of AKI as well as non-early recovery AKI; however, there was no difference in the amount of colloid per body weight administered between the groups. Although this result should be generalized with caution due to the small size of the study population, previous meta-analyses, and narrative reviews have reported that a high volume of intraoperative hydroxyethyl starch increases the risk of AKI after lung transplantation^5,6^. Regarding long-term renal function after lung transplantation, attention needs to be paid to the higher overall incidence of CKD (47%), indicating that kidney injury is not a one-time event but is a continuous process in this subset of patients.

Concerning the primary endpoint of this study, a remarkable decrease in DAOH-90 was observed in non-early recovery AKI group compared with no-AKI group, while early recovery AKI group have similar DAOH-90 to those of the no-AKI group. DAOH, a composite patient-centered outcome, emerged as the most desirable outcome for patients—being free from complications and readmissions, and returning to normal life promptly^19^. Compared with mid- and long-term mortality endpoint in patients undergoing major surgery, DAOH accounts for multiple outcome parameters reflecting the days spent healthily after surgery, and its use has been augmented. In such context, DAOH after lung transplantation could be a valuable metric to assess postoperative quality of life and recovery, while it has received limited attention. In previous studies, a prolonged hospitalization after lung transplantation not only increased the risk of early complications, such as *C. difficile* infection, but also showed a strong association with late survival^27^, and shorter DAOH was also related to higher 1-year mortality after surgery^28^, all of which indicated a long-term prognostic value of DAOH.

In the present study, the occurrence of AKI, especially non-early recovery AKI, significantly shortened DAOH-90. AKI per se is reported to increases the length of hospital stay, and also through the contribution to the development of PGD and prolonged mechanical ventilation after lung transplantation^9,29^. Of interest, the incidence of grade 3 PGD within 72 h and failure to achieve weaning from mechanical ventilation within POD 7 were higher only in the non-early recovery AKI group and not in the early recovery AKI group compared with the no-AKI group. By extension, a significantly shorter DAOH-90 was observed only in the non-early recovery AKI group and not in the early recovery AKI group. Moreover, multivariable quantile regression revealed non-early recovery AKI as an independent risk factor of shorter DAOH-90, even when adjusting for other major confounders. Our results align with previous literatures showing that AKI recovery subgroups differently affect long-term outcomes^11,13,16^. These results indicate the necessity of risk stratification and close monitoring of patients susceptible to prolonged manifestation of AKI in the management of lung transplantation patients to achieve clinical goals of being able to return to life outside the hospital and recovery of quality of life. Besides AKI, patients who received tracheostomy before lung transplantation were independently experienced shorter DAOH-90, many of whom often required mechanical ventilation preoperatively, which was an independent predictor of prolonged length of hospital stay after lung transplantation in the previous study^27^.

Regarding the long-term influence of AKI, the 1-year mortality rate and Kaplan–Meier 1-year survival curve showed significant differences only between the no-AKI and non-early recovery AKI groups in this study and were worse in the non-early recovery AKI group. Notably, CKD progression was most prominent in the non-early AKI group (even when compared with the early recovery AKI group), which subsequently led to increased 1-year mortality (Fig. 4). These results further highlighted the importance of early recovery AKI in improving the postoperative quality of life in patients undergoing lung transplantation.

In terms of AKI stages, out of 86 patients with AKI, 46 (53%) had stage 1 AKI, of which 30 (65%) achieved early recovery, and 20 (23%) had stage 3 AKI, of which only 2 (10%) achieved early recovery. The median DAOH-90 in patients with stage 3 AKI was 0 in contrast to 27 days in patients with stage 1 AKI (*P* = 0.01). Unlike the differences in recovery type and DAOH-90 according to the AKI stages, there were no differences in the incidence of CKD (*P* = 0.417). Thus, it can be considered that not only the stages of AKI but also the recovery status should be emphasized in the longitudinal assessment of renal dysfunction. These results were further supported by the lack of difference in the serially assessed serum creatinine levels up to 1-year between the no-AKI and early recovery AKI groups after POD 5.

This study had some limitations. First, there were inherent limitations related to being a single-centered, retrospective design. Second, like in many other studies, urine output criteria were not used for AKI diagnosis because these data were unavailable^6,9^. This may have led to the underestimation of the incidence of AKI^30^. Third, DAOH-30 has been proposed as a more suitable index in elective surgeries, considering the confounding influence of postoperative mortality^19,28^. However, DAOH-30 in our study population was 0, and to account for the high-risk of lung transplantation, DAOH-90 was a more suitable index of patient-centered outcome in this subset of patients.

In conclusion, the current study first demonstrated that AKI significantly worsened patient recovery in terms of both patient-centered (DAOH-90) and clinical outcome (CKD progression and 1-year mortality) measures. Moreover, the non-early recovery AKI group exhibited the worst prognosis in terms of DAOH-90, CKD progression, and 1-year mortality, highlighting the important role of AKI and its early-recovery on both the quality of life and clinical outcomes in patients receiving lung transplantation.

## Supplementary Information

Supplementary Information.

## Data Availability

The data analyzed during the current study are available from the corresponding author upon reasonable request.

## Abbreviations

AKI: Acute kidney injury
CKD: Chronic kidney disease
DAOH: Days alive out of hospital
DAOH-90: Days alive out of hospital within the first 3 months
ECMO: Extracorporeal membrane oxygenation
ICU: Intensive care unit
LOS: Length of stay
PGD: Primary graft dysfunction
POD: Postoperative day

## References

[1] Abidi, Y. *et al.* Lung transplant rehabilitation: A review. *Life (Basel)* **13**, 506 (2023). 36836863 10.3390/life13020506 PMC9962622

[2] Hoffman, M., Chaves, G., Ribeiro-Samora, G. A., Britto, R. R. & Parreira, V. F. Effects of pulmonary rehabilitation in lung transplant candidates: A systematic review. *BMJ Open* **7**, e013445 (2017). 28159852 10.1136/bmjopen-2016-013445 PMC5294003

[3] Hume, E. *et al.* Exercise training for lung transplant candidates and recipients: A systematic review. *Eur Respir Rev* **29**, 200053 (2020). 33115788 10.1183/16000617.0053-2020 PMC9488968

[4] M’Pembele, R. *et al.* Life impact of VA-ECMO due to primary graft dysfunction in patients after orthotopic heart transplantation. *ESC Heart Fail.* **9**, 695–703 (2022). 34734490 10.1002/ehf2.13686 PMC8788039

[5] Jing, L. *et al.* Acute kidney injury after lung transplantation: a narrative review. *Ann. Transl. Med.* **9**, 717 (2021). 33987415 10.21037/atm-20-7644 PMC8106087

[6] Doricic, J. *et al.* Kidney injury after lung transplantation: Long-term mortality predicted by post-operative day-7 serum creatinine and few clinical factors. *PLoS One* **17**, e0265002 (2022). 35245339 10.1371/journal.pone.0265002 PMC8896732

[7] Chan, E. G. *et al.* Postoperative acute kidney injury and long-term outcomes after lung transplantation. *Ann. Thorac. Surg.* **116**, 1056–1062 (2023). 37414386 10.1016/j.athoracsur.2023.06.016

[8] Lertjitbanjong, P. *et al.* Acute kidney injury after lung transplantation: A systematic review and meta-analysis. *J. Clin. Med.* **8**, 1713 (2019). 31627379 10.3390/jcm8101713 PMC6833042

[9] Scaravilli, V. *et al.* Longitudinal assessment of renal function after lung transplantation for cystic fibrosis: Transition from post-operative acute kidney injury to acute kidney disease and chronic kidney failure. *J. Nephrol.* **35**, 1885–1893 (2022). 35838909 10.1007/s40620-022-01392-z PMC9458565

[10] Andrew, B. Y. *et al.* Identification of trajectory-based acute kidney injury phenotypes among cardiac surgery patients. *Ann. Thorac. Surg.* **114**, 2235–2243 (2022). 34968444 10.1016/j.athoracsur.2021.11.047 PMC9237188

[11] Kellum, J. A., Sileanu, F. E., Bihorac, A., Hoste, E. A. & Chawla, L. S. Recovery after acute kidney injury. *Am. J. Respir. Crit. Care Med.* **195**, 784–791 (2017). 27635668 10.1164/rccm.201604-0799OC PMC5363967

[12] Bhatraju, P. K. *et al.* Acute kidney injury subphenotypes based on creatinine trajectory identifies patients at increased risk of death. *Crit. Care* **20**, 372 (2016). 27852290 10.1186/s13054-016-1546-4 PMC5112626

[13] Bhatraju, P. K. *et al.* Integrated analysis of blood and urine biomarkers to identify acute kidney injury subphenotypes and associations with long-term outcomes. *Am. J. Kidney Dis.* **82**, 311-321e311 (2023). 37178093 10.1053/j.ajkd.2023.01.449 PMC10523857

[14] Lameire, N. H. *et al.* Harmonizing acute and chronic kidney disease definition and classification: Report of a kidney disease—improving global outcomes (KDIGO) consensus conference. *Kidney Int.* **100**, 516–526 (2021). 34252450 10.1016/j.kint.2021.06.028

[15] Forni, L. G. *et al.* Renal recovery after acute kidney injury. *Intensive Care Med.* **43**, 855–866 (2017). 28466146 10.1007/s00134-017-4809-x PMC5487594

[16] Bhatraju, P. K. *et al.* Association between early recovery of kidney function after acute kidney injury and long-term clinical outcomes. *JAMA Netw. Open* **3**, e202682–e202682 (2020). 32282046 10.1001/jamanetworkopen.2020.2682 PMC7154800

[17] Bhatraju, P. K. *et al.* Acute kidney injury subphenotypes based on creatinine trajectory identifies patients at increased risk of death. *Crit. Care* **20**, 1–10 (2016). 27852290 10.1186/s13054-016-1546-4 PMC5112626

[18] Snell, G. I. *et al.* Report of the ISHLT Working group on primary lung graft dysfunction, part I: Definition and grading—a 2016 consensus group statement of the international society for heart and lung transplantation. *J. Heart Lung Transplant.* **36**, 1097–1103 (2017). 28942784 10.1016/j.healun.2017.07.021

[19] Myles, P. S. *et al.* Validation of days at home as an outcome measure after surgery: A prospective cohort study in Australia. *BMJ Open* **7**, e015828 (2017). 28821518 10.1136/bmjopen-2017-015828 PMC5629653

[20] Huang, L., Frandsen, M. N., Kehlet, H. & Petersen, R. H. Days alive and out of hospital after enhanced recovery video-assisted thoracoscopic surgery lobectomy. *Eur. J. Cardiothorac. Surg.*10.1093/ejcts/ezac148 (2022). 35234866 10.1093/ejcts/ezac148

[21] Van Slambrouck, J. *et al.* A focused review on primary graft dysfunction after clinical lung transplantation: A multilevel syndrome. *Cells* **11**, 745 (2022). 35203392 10.3390/cells11040745 PMC8870290

[22] Koenker, R. *Quantile Regression* (Cambridge University Press, 2005).

[23] Koenker, R. Quantreg: Quantile regression. R package version 5.97 (2023). http://CRAN.R-project.org/package=quantreg (2023).

[24] Allaire, J. *et al.* Package ‘networkD3’. *D3 JavaScript network graphs from R* (2017).

[25] Otto, E. *et al.* Overview of Sankey flow diagrams: Focusing on symptom trajectories in older adults with advanced cancer. *J. Geriatr. Oncol.* **13**, 742–746 (2022). 35000890 10.1016/j.jgo.2021.12.017 PMC9232856

[26] Boyer, N., Eldridge, J., Prowle, J. R. & Forni, L. G. Postoperative acute kidney injury. *Clin. J. Am. Soc. Nephrol.* **17**, 1535–1545 (2022). 35710717 10.2215/CJN.16541221 PMC9528271

[27] Banga, A. *et al.* Hospital length of stay after lung transplantation: Independent predictors and association with early and late survival. *J Heart Lung Transplant.* **36**, 289–296 (2017). 27642060 10.1016/j.healun.2016.07.020

[28] Spurling, L. J., Moonesinghe, S. R. & Oliver, C. M. Validation of the days alive and out of hospital outcome measure after emergency laparotomy: A retrospective cohort study. *Br. J. Anaesth.* **128**, 449–456 (2022). 35012739 10.1016/j.bja.2021.12.006

[29] Tagawa, M. *et al.* Acute kidney injury as an independent predictor of infection and malignancy: The NARA-AKI cohort study. *J. Nephrol.* **32**, 967–975 (2019). 31617159 10.1007/s40620-019-00662-7

[30] Quan, S. *et al.* Prognostic implications of adding urine output to serum creatinine measurements for staging of acute kidney injury after major surgery: A cohort study. *Nephrol. Dial. Transplant.* **31**, 2049–2056 (2016). 27941063 10.1093/ndt/gfw374

