# Supplementary material for: Influence of acute kidney injury and its recovery subtypes on patient-centered outcomes after lung transplantation
Source: Sci Rep. 2024 May 7;14:10480. doi: 10.1038/s41598-024-61352-4 (PMC11076280; doi:10.1038/s41598-024-61352-4)
Supplement: Supplementary file 1 — Supplementary Information. [file 41598_2024_61352_MOESM1_ESM.docx]

Supplementary Information

- Supplementary Methods.
- Supplementary Table 1-2

Supplementary Methods

Statistical analysis

In addition, the patient characteristics associated with AKI status and AKI recovery groups (the main exposures of interest) were examined in an exploratory manner. As a large number of demographic, clinical, and pre/intraoperative variables were available, the least absolute shrinkage and selection operator (LASSO)^1^ feature selection was used to evaluate relative variable importance, and backward elimination via either statistical significance or the Akaike Information Criterion (AIC) was used to determine the final list of variables that were significantly associated with either AKI status or AKI recovery groups (Table S1 and S2).

Supplementary Tables

Table S1. Multivariable logistic regression results of patient characteristics associated with AKI occurrence (Yes/No) among lung transplantation patients.

| Variable | odds ratio | 95% CI | P value |
| --- | --- | --- | --- |
| female | 1.780 | 1.007-3.147 | 0.047 |
| preoperative neutrophil count | 0.910 | 0.846-0.979 | 0.011 |
| preoperative MV status | 2.074 | 1.122-3.832 | 0.020 |
| intraoperative ECMO weaning | 0.545 | 0.306-0.969 | 0.039 |
| intraoperative use of colloid | 2.104 | 1.191-3.716 | 0.010 |

AKI, acute kidney injury; CI, confidential interval; ECMO, extracorporeal membrane oxygenation; MV, mechanical ventilation

Table S2. Multivariable multimodal logistic regression results of patient characteristics associated with AKI recovery group^*^ among lung transplantation patients

| Variable | AKI recovery group* | Odds Ratio | 95% CI | P value |
| --- | --- | --- | --- | --- |
| preoperative tracheostomy status | 1 (vs. 0) | 1.061 | 0.472-2.387 | 0.886 |
|  | 2 (vs. 0) | 2.878 | 1.418-5.842 | 0.003 |
| intraoperative use of colloid | 1 (vs. 0) | 1.555 | 0.764-3.164 | 0.223 |
|  | 2 (vs. 0) | 2.577 | 1.290-5.148 | 0.007 |

AKI, acute kidney injury; CI, confidential interval

^*^0, no-AKI group; 1, early recovery AKI group; 2, non-early recovery AKI group

References

1. Tibshirani, R. Regression shrinkage and selection via the lasso. *Journal of the Royal Statistical Society Series B: Statistical Methodology* **58**, 267-288 (1996).
